# Intractable hiccups (*singultus*) abolished by risperidone, but not by haloperidol

**DOI:** 10.1186/s12991-015-0051-5

**Published:** 2015-03-05

**Authors:** Tadashi Nishikawa, Yoichiro Araki, Teruo Hayashi

**Affiliations:** Seiwakai Nishikawa Hospital, 293-2 Minato-machi, Hamada, Shimane 697-0052 Japan

**Keywords:** Hiccups, Dopamine, Serotonin, Antipsychotic, Risperidone

## Abstract

Hiccups or *singulata* are rhythmic involuntary movements of the diaphragm, caused by a variety of conditions that interfere with the functions of the nerve nuclei in the medulla and supra-spinal hiccup center. Although neurotransmitters and receptors involved in the pathophysiology of hiccups are not defined well, dopamine has been considered to play an important role. In some cases, chlorpromazine or other antipsychotics are used for the treatment of intractable hiccups but their efficacy is often limited. This report involves an 18-year-old patient who experienced two episodes of intractable hiccups triggered by stress, which lasted for weeks or even months. In both episodes, haloperidol was initially used, but there was no significant effect. In contrast, risperidone, the second-generation antipsychotic that possesses a dopamine-serotonin antagonist property, completely abolished the hiccups 6 hours after administration. This is one of few case reports in which two antipsychotics were challenged for a single patient with hiccups, and the effects of the drugs were obviously different. Our finding suggests that, in addition to dopaminergic system, the serotonergic systems may be involved in the pathophysiology of some hiccup cases and that the serotonin-acting antipsychotics such as risperidone should be considered as a choice in the drug treatment of intractable hiccups.

## Background

Hiccups (*singultus*) are involuntary contractions of the diaphragm that repeat several times per minutes with “hic” sounds at the pharynx [1,2]. Hiccups are caused by a variety of conditions that interfere with the functions at the nerve nuclei in the medulla and/or the supra-spinal hiccup center, which regulate the rhythmic movement of the diaphragm [3,4]. It is not well defined which kinds of neurotransmitters and receptors in these nervous systems are involved in the pathophysiology of hiccups, but dopamine has been considered to play a role [2,5].

Hiccups are very common physical responses and are usually short lasting and self limiting [1,2]. However, medical treatments are sometimes required for persistent or intractable hiccups. Chlorpromazine and metoclopramide are the most frequently used first-line treatments [2,3]. Assuming that dopamine is involved in the pathophysiology of hiccups, chlorpromazine and other antipsychotics should provide clear outcomes in the treatment of hiccups [2,6]. However, these drugs do not always provide favorable results. Furthermore, recent case reports suggest that antipsychotics may actually trigger hiccups in some cases [7-9]. So it seems that, so far, no pharmacotherapeutic algorithm in treatment of hiccups has been established.

Here, we report a case of persistent hiccups abolished after the administration of risperidone but not after the administration of haloperidol.

## Case presentation

Our patient is an 18-year-old male. When he was 16 and when he was busy for school activities, he suffered from persistent and rhythmic hiccup-like breaths that often disturbed his speech. The symptoms, however, disappeared in sleep. Initially, he consulted an otorhinolaryngologist, but no diagnostic conclusion was made. At one department of pediatrics of a university hospital, vocal tic disorder was suspected and he was administered with a daily dose of haloperidol 1.5 mg and trihexyphenidyl 4 mg. After 3 months, the symptom had spontaneously disappeared. Thus, the contribution of haloperidol treatment to the outcome was unclear.

After graduating high school, he started a job at a local company. After getting into that company, he attended a trainee seminar at the age of 18, which was located far from his hometown. There, he suffered from upper airway inflammation, with symptoms such as: coughs, sputum, and slight fevers. He was admitted to a general hospital for 2 days, and these symptoms disappeared. After the discharge, however, he began to complain about rhythmic hiccup-like breaths similar to those he experienced while he was in high school. An internist related his involuntary movement to his previous diagnosis, i.e., vocal tic disorder. Antitussive drugs, haloperidol 1.5 mg and trihexyphenidyl 4 mg, were administered again for 16 days. Since his symptoms were not responsive to the medication, he consulted to our hospital.

His body temperature was normal. All laboratory data including chest X-ray and C-reactive protein were normal. Pharyngeal noises that sounded like “gyu, gyu” were heard almost every second. The noises continued all day long but disappeared while the patient was sleeping. To stop his hiccups, he tried a variety of physical measures, such as holding breath and drinking water while pinching the nose. But, none of them was effective. At the medical inspection of his body muscle movements, we found a series of breathing diaphragm spasms and twitching of his abdominal muscles. Those movements synchronized well with his pharyngeal noises. By gathering these laboratory and observational data together, we concluded that he suffered from hiccups but not from cough or vocal tic disorder. Diazepam 10 mg was intramuscularly administered, but no apparent effect was observed. Next, we decided to challenge an atypical antipsychotic drug. For monitoring of the drug efficacy, we chose risperidone solution at a relatively high dose that enables the rapid increase of the blood concentration of the drug. In contrast to diazepam, oral solution of risperidone 3 mg (3 ml) began to decrease his hiccup rates 15 minutes after the administration. For the follow-up monitoring of his hiccups, he was hospitalized to our hospital. Six hours after the first administration of risperidone solution, complete abolishment of his hiccups was observed. He could talk smoothly without any pharyngeal noises and twitches of the abdominal muscles. He claimed that the symptoms observed in his two hiccup episodes were exactly the same and that the episodes occurred when busy and stressful days continued. Next day, he was discharged without any symptoms observed. Risperidone was not re-administered. Three psychiatrists found no apparent signs supporting his diagnosis of schizophrenia, depression, personality disorders, and anxiety or somatoform disorders.

## Conclusion

Cough and intense emotions such as fear and anxiety are known to be causes of hiccups [9-11]. It is likely that coughs due to pre-existing colds and stresses during the training program caused intractable hiccups in our case that has no comorbidity of psychiatric disorders. It would be particularly important to highlight that his hiccups dramatically responded to risperidone but not to haloperidol, in spite of the fact that both are the potent inhibitors of dopamine D_2_ receptors. The difference in the drug effect could be partly due to the fact that haloperidol was co-administered with trihexyphenidyl. Muscarinic receptor antagonist trihexyphenidyl is known to increase the extracellular dopamine and gamma-aminobutyric acid (GABA) levels in the substantia nigra [12]. However, whether the elevation of dopamine or GABA levels in the substantia nigra affects involuntary movements of the diaphragm is unknown. Another possibility explaining the effect of risperidone could be the different receptor-binding profiles of these two antipsychotics. Risperidone possesses slightly lower or similar affinities for dopamine D_1_, D_2_, D_3_, and D_4_ receptors when compared with haloperidol [13-16]. On the other hand, risperidone possesses a much higher affinity for the serotonin 2A (5-HT_2A_) receptors than haloperidol, thus the D_2_/5-HT_2A_ affinity ratio of risperidone is approximately 500 times higher than that of haloperidol [13,16,17]. In addition to 5-HT_2A_ inhibition, risperidone is known to stimulate cortical and subcortical serotonin output [18]. 5-HT is known to be involved in both the anxiety and the neural transmission in the reflex arcs, which are involved in the generation of hiccups [3,9]. Furthermore, recent reports are accumulating evidence that serotonergic agents may ameliorate hiccups [19,20]. These findings, together with our case, indicate that serotonin-acting antipsychotics such as risperidone may be effective in the treatment of some hiccup cases that may involve dysfunctions of the serotonergic systems (e.g., hiccups related to stress or anxiety). Furthermore, our finding may suggest that there are subtypes of intractable hiccups and the associated comorbidity/etiology may underlie different sensitivity to a specific category of antipsychotic drugs.

## Consent

Written informed consent was obtained from the patient for publication of this case report and any accompanying images. A copy of the written consent is available for review by the Editor-in-Chief of this journal.
